# Role of Adipokines in the Development of Metabolic Syndrome in Patients With Polycystic Ovary Syndrome

**DOI:** 10.7759/cureus.82355

**Published:** 2025-04-16

**Authors:** Chaitali Maitra, Arjun Maitra

**Affiliations:** 1 Biochemistry, Dr. S.S. Tantia Medical College Hospital and Research Centre, Sri Ganganagar, IND; 2 Physiology, Dr. S.S. Tantia Medical College Hospital and Research Centre, Sri Ganganagar, IND

**Keywords:** adiponectin, leptin, metabolic syndrome, obesity, resistin

## Abstract

Introduction

Patients with polycystic ovary syndrome (PCOS) have a higher risk of developing metabolic syndrome (MetS), as similarities in pathophysiology exist between the two. Adipokines are the key molecules that alter the adipose tissue metabolism and distribution, leading to MetS. This study was conducted to assess the levels of adipokines among patients with PCOS with and without MetS and to establish the role of adipokines as an early predictor for the development of MetS in PCOS.

Materials and methods

In this monocentric, tertiary hospital-based study, 144 patients with PCOS were selected and classified according to Rotterdam criteria and screened for MetS. Patients were grouped into MetS and non-MetS categories based on the presence of MetS. Adipokine levels (adiponectin, leptin, and resistin) were measured and compared, along with other biochemical and anthropological parameters. Data were analyzed using Jamovi statistical software, version 2.3 (Retrieved from https://www.jamovi.org), using an independent samples t-test and multinomial regression analysis.

Results

Out of the 144 patients included in the study, 58 were diagnosed with MetS and grouped as MetS. The prevalence of MetS was maximum among the non-PCO (O+HA) phenotype (53 out of 58). Significant differences (p<0.001) in adipokines were observed between the MetS and non-MetS groups. Adiponectin, leptin, and resistin were found to have a significant role as predictors of MetS at an early stage of development of the syndrome, along with other common predictors. However, in later stages, fasting, blood glucose, waist circumference, and triglycerides remain significant predictors.

Conclusion

The present study reports a high prevalence of MetS among PCOS cases (40.27%). Altered adipokine levels may be significantly associated with the early stages of MetS development and may be used as an early diagnostic tool for the management of MetS in PCOS. Therefore, the measurement of adipokines during preliminary investigations may be used as an early diagnostic tool for the management of MetS in PCOS.

## Introduction

Metabolic syndrome (MetS) is a syndrome characterized primarily by hypertension, hyperglycemia, hyperinsulinemia, and dyslipidemia. MetS is also established as a strong risk factor for cardiovascular diseases (CVD). It was also observed that in the absence of diabetes, there was an association of MetS with coronary heart disease. Consequently, researchers concluded that MetS is a cluster of risk factors for both diabetes and CVD [1]. Obesity is another factor associated with MetS, but it is the central fat that is the predictor. Other factors considered contributory to the pathophysiology of MetS are endothelial dysfunction, lifestyle, and genetics [2].

Commonalities of features exist between polycystic ovary syndrome (PCOS) and MetS. In both syndromes, peripheral insulin resistance and compensatory hyperinsulinemia are observed. However, obesity is a common factor in both type 2 diabetes mellitus (T2DM) and cardiovascular disease (CVD) [3]. Among the individual factors for the diagnosis of MetS, decreased high-density lipoprotein (HDL) followed by increased waist circumference (WC) and increased triglyceride (TG) levels are also widely prevalent in PCOS [4].

Various studies have demonstrated that PCOS has a higher risk of developing MetS in patients who are overweight and obese. It has also been reported that it is the metabolic markers that contribute to the heterogeneity of prevalence of MetS in PCOS rather than markers of reproductive dysfunction [5]. The reported prevalence of MetS in PCOS varies among the population. Glueck et al. (2003) showed that 43% of Caucasian PCOS patients have features of MetS [6]. A study by Essah and Nestler (2006) indicated that 43% of PCOS have MetS in the US population [7]. A study among the Iranian population reported a prevalence of 28.8% of MetS in PCOS [8]. Brazilian women showed a prevalence of 28.4% [9]. A 24.9% prevalence was reported in the Hong Kong Chinese population [10]. The overall prevalence of MetS among PCOS subjects was reported as 37.5% in a study conducted in the southern state of India [11]. A cross-sectional multicentric study reported a prevalence of 35.07% in Indian women with PCOS, which is independent of obesity [12].

All phenotypes defined by the Rotterdam criteria (defined by the presence of two out of three of the following criteria: oligo (O) or anovulation, hyperandrogenism (HA), and polycystic (P) ovarian morphology) showed the presence of MetS in varied degrees [13]. Maximum prevalence is shown by the non-PCO phenotype (O+HA) along with insulin resistance (IR), followed by the classical phenotype (P+O+HA). Normo-androgenic (P+O) and ovulatory phenotype (P+HA) have a lower prevalence of MS [14]. In contrast, the South Asian population showed the highest prevalence of MetS in the classical phenotype (P+O+HA), followed by the hyperandrogenic one. In the remaining two phenotypes (P+O and P+HA), the prevalence was low. Among the phenotypes, the only component of MetS that showed variation was WC [15]. It is not HA but obesity emerged as a significant predictor of MetS in the PCOS population.

The risk of T2DM, hypertension, and CVD increases with an increase in visceral fat and WC. WC has been proven as a good indicator of obesity-related diseases [16]. PCOS women also show increased visceral fat when compared to their BMI-matched controls [17]. Visceral adipose tissue has become a key determinant of metabolic risk in terms of secretion of proinflammatory adipokines [18].

Leptin is the key factor controlling adipose tissue expansion. In the case of energy surplus due to downregulation of leptin receptors in adipose tissue, free fatty acid oxidation is attenuated, but fat accumulation continues [19]. Increased leptin and decreased adiponectin are considered characteristic of patients with T2DM and MetS [20].

Adiponectin increases fatty acid oxidation and glycolysis through receptors AdipoR1 and hepatic AdipoR2 by activation of two signaling pathways, AMPK (AMP-activated protein kinase) and PRAR-α (peroxisome proliferator-activated receptor) cascade, respectively [21]. Polymorphism of the gene encoding adiponectin (*ADIPOQ*) is shown to be associated with low circulating levels of adiponectin, IR, and obesity [22].

Resistin has been considered one of the proinflammatory cytokines, and this fact is supported by the presence of higher levels of resistin mRNAs in human monocytes [23]. Resistin also affects intracellular signaling pathways. Upregulation of its expression in T2DM by human pancreatic cells has been reported [24]. The role of resistin has also been explored in the development of MetS components [25].

The aim of this study is to investigate the adipokines in PCOS with and without the expression of MetS and establish the role of adipokines as early predictors for the development of MetS in PCOS.

## Materials and methods

The study subjects were recruited from patients reporting menstrual irregularity and infertility to the outpatient department of Obstetrics and Gynecology, Mayo Institute of Medical Sciences, Barabanki. The study was conducted between November 2019 and October 2021. A total of 144 participants were ultimately recruited for the study, having met our inclusion criteria and been diagnosed with PCOS according to the Rotterdam criteria. Individuals under 18 years and over 40 years of age and those diagnosed with late-onset congenital adrenal hyperplasia, thyroid disorders, hyperprolactinemia, or androgen-secreting neoplasms were excluded from the study. Participants who were lactating or pregnant were excluded from the study. Additionally, participants utilizing a hormonal intrauterine device or undergoing medication (including oral contraceptives) were excluded from the study. The study was approved by the institutional ethics committee of the Mayo Institute of Medical Sciences (approval no.: MIMS/Ex/2019/199 dated 19/11/2019). Informed consent was obtained from all participants.

The subjects underwent different radiological, biochemical, and hormonal investigations as per the examination protocol. Data for anthropometric parameters (weight, height, and waist and hip circumference) were collected, and BMI was calculated using a standard formula. The waist measurement was taken with non-stretchable tape after a normal expiration when subjects were standing, and weight was evenly distributed on both feet. An average of three measurements was taken to calculate the mean systolic and diastolic blood pressure (BP) by an automatic sphygmomanometer (Omron HEM 711_C1, Omron Healthcare, Kyoto, Japan).

Fasting venous blood samples were collected for biochemical and hormonal parameters. Glucose and TG levels were determined by enzymatic assay. HDL was estimated by direct assay based on coupled precipitation and selective reaction of HDL. All adipokines, insulin, and testosterone were estimated by enzyme-linked immunosorbent assay. Total testosterone was quantitatively estimated using the enzyme-linked immunosorbent assay (ELISA) kit DiaMetra (cat. no.: DKO002, RRID: AB_3075391; Perugia, Italy), and insulin was estimated using ELISA kit DiaMetra (cat. no.: DKO076, RRID: AB_3075466). Adiponectin was estimated based on a sandwich assay by ELISA kit Demeditec Diagnostics (cat. no.: DEE009, RRID: AB_3075465; Kiel, Germany). Resistin was determined by the resistin (RETN) ELISA kit from Sincere Biotech (cat. no.: E13651978; Beijing, China), and leptin was measured using the ELISA kit from Demeditec Diagnostics (cat. no.: DEE007, RRID: AB_3075464).

To estimate the presence of MetS, the NCEP ATP III (National Cholesterol Education Program's Adult Treatment Panel) definition was considered, which recommends the presence of a higher value than the cutoff level of at least three of five parameters, which include WC, fasting blood sugar (FBS), TG levels, HDL, and BP. The modified ATP III (2005) criteria were used for BP, WBC, and FBS to account for ethnic differences. The following cutoff values were considered: WC (≥80 cm for females), FBS (≥100 mg/dL), TG (≥150 mg/dL), HDL (<50 mg/dL), and BP (>135/85 mmHg or antihypertensive treatment) [26].

Statistical analyses were performed using Jamovi statistical software, version 2.3 (Retrieved from https://www.jamovi.org). Independent samples t-tests were employed to compare between groups, and multinomial regression analysis was used to assess the predictivity of the different parameters of MetS. Statistical significance for all analyses was defined as a p-value of less than 0.05.

## Results

**Figure 1:**
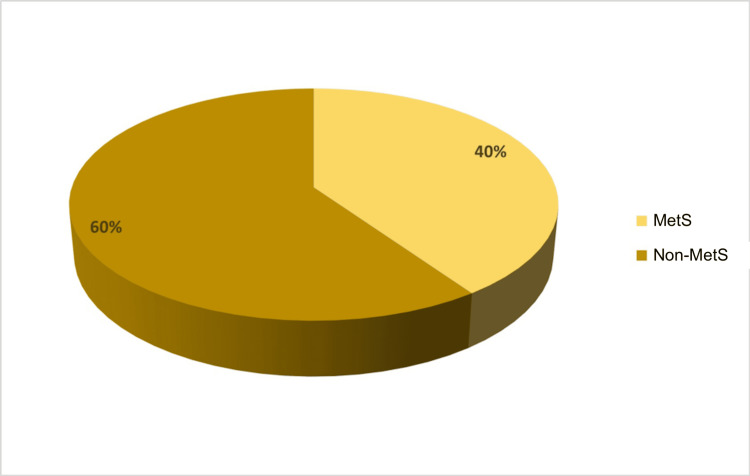
Presence of MetS and non-MetS among the studied PCOS patients MetS: PCOS patients with metabolic syndrome; non-MetS: PCOS patients without metabolic syndrome; PCOS: polycystic ovary syndrome

This study included 144 individuals diagnosed with PCOS. Among these participants, 58 were identified as having MetS, and 86 did not fulfill the MetS criteria (Table 1, Figure 1).

**Table 1:** Presence of MetS among the studied PCOS patients MetS: PCOS patients with metabolic syndrome; non-MetS: PCOS patients without metabolic syndrome; PCOS: polycystic ovary syndrome

Category	Frequency	Percentage	Valid Percentage	Cumulative Percentage
MetS	58	40.27778	40.27778	40.27778
Non-MetS	86	59.72222	59.72222	100
Total	144	100.0	100.0	-

**Figure 2:**
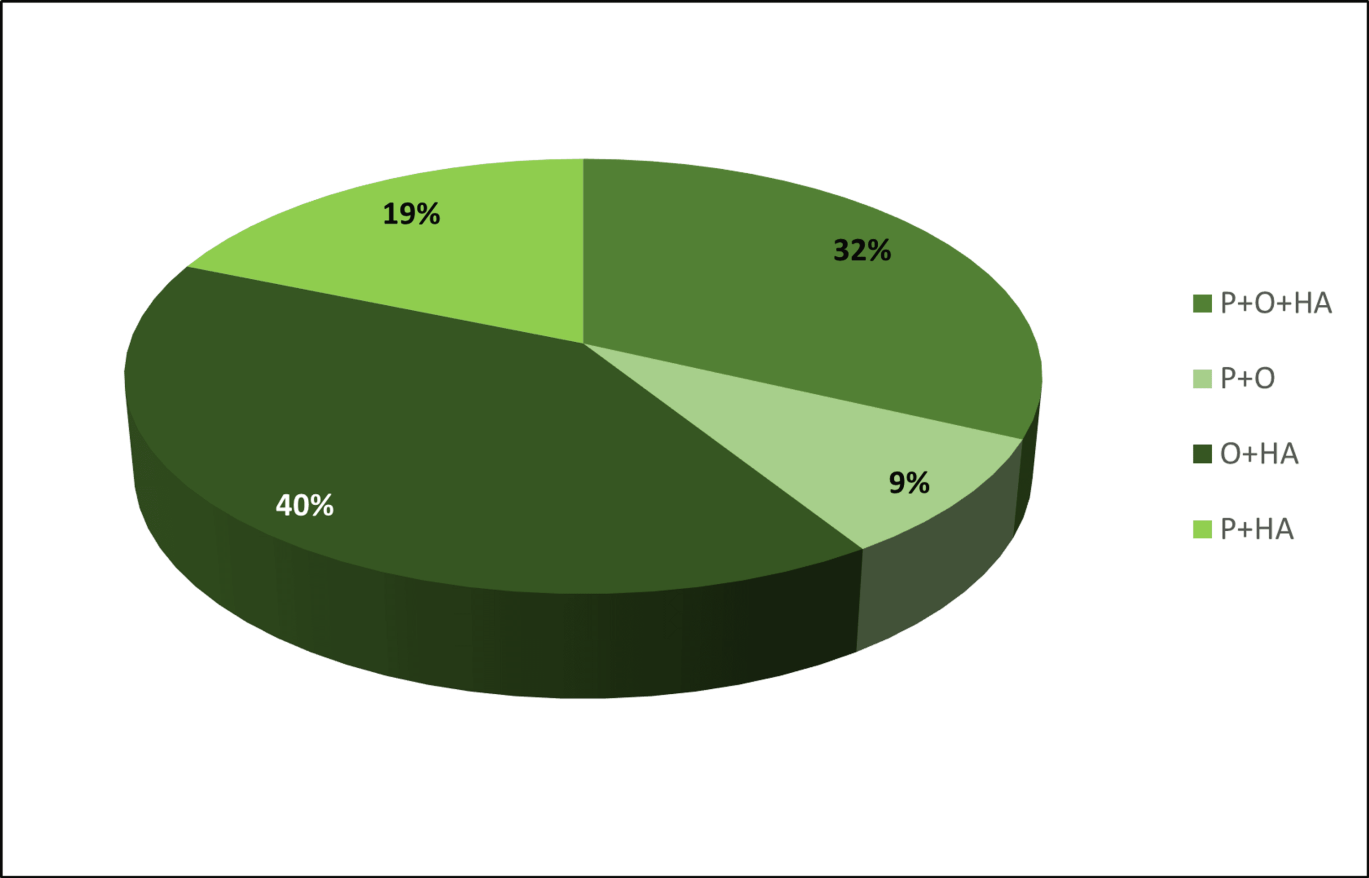
Distribution of PCOS phenotypes O: oligo or anovulation; HA: hyperandrogenism; P: polycystic; PCOS: polycystic ovary syndrome

Patients belonging to the P+O (n = 13) and P+HA (n = 27) phenotypes did not exhibit MetS, whereas 53 out of 58 patients belonging to the O+HA phenotype had MetS. Among the complete PCOS (P+O+HA) phenotype, five out of 46 patients fulfilled the criteria of MetS (Table 2, Figure 2).

**Table 2:** Distribution of PCOS phenotypes and MetS among the four different phenotypes O: oligo or anovulation; HA: hyperandrogenism; P: polycystic; MetS: metabolic syndrome; PCOS: polycystic ovary syndrome

Phenotypes	Total Count	% PCOS Study Population (n=144)	MetS	% MetS	% Without MetS
P+O+HA	46	31.94	5	10.87	89.13
P+O	13	9.03	0	0	100
O+HA	58	40.28	53	91.38	8.62
P+HA	27	18.75	0	0	100

The baseline characteristics of the studied PCOS subjects (n = 144) in the PCOS with MetS group and without MetS group were described in Table 3. Table 4 shows that BMI, WC, hip circumference, and waist-to-hip ratio were found to be significantly (p < 0.05) high among the PCOS subjects with MetS. A significantly low adiponectin level (7.19 ± 2.02 µg/mL) was observed among the subjects suffering from MetS, whereas leptin (12.11 ± 1.71 ng/mL) and resistin (6.33 ± 0.83 ng/mL) levels were significantly (p < 0.05) high among the subjects with MetS. Serum insulin and testosterone levels were also significantly high among the subjects with MetS (Table 5). FBS, TG, and cholesterol levels were significantly high among the MetS group, and HDL-C was observed to be significantly high among the non-MetS group (Table 6).

**Table 3:** Baseline characteristics of the studied PCOS patients Data are expressed as mean ± SD. SD: standard deviation; BMI: body mass index; WHR: waist-to-hip ratio; FBS: fasting blood sugar; HDL-C: high-density lipoprotein cholesterol; SBP: systolic blood pressure; DBP: diastolic blood pressure

Variables	PCOS (n=144)	Non-MetS (n=58)	MetS (n=86 )
Age (years)	27.46 ± 4.03	27.18 ± 3.46	27.87 ± 4.76
BMI	26.374 ± 3.19	24.34 ± 1.48	29.39 ± 2.62
Waist (cm)	92.764 ± 5.73	89.94 ± 5.61	96.94 ± 2.36
Hip (cm)	99.728 ± 2.52	98.99 ± 2.75	100.82 ± 1.60
WHR	0.930 ± 0.04	0.90 ± 0.05	0.96 ± 0.02
Adiponectin (μg/mL)	10.281 ± 3.27	12.35 ± 2.10	7.19 ± 2.02
Resistin (ng/mL)	5.412 ± 1.09	4.78 ± 0.74	6.33 ± 0.83
Leptin (ng/mL)	10.946 ± 2.14	10.15 ± 2.04	12.11 ± 1.71
FBS (mg/dL)	90.204 ± 9.05	86.51 ± 8.08	95.67 ± 7.55
Triglyceride (mg/dL)	144.812 ± 19.19	131.46 ± 9.63	164.60 ± 10.90
Cholesterol (mg/dL)	162.067 ± 15.60	153.71 ± 11.91	174.44 ± 11.76
HDL-C (mg/dL)	38.494 ± 2.27	39.04 ± 2.37	37.67 ± 1.83
SBP (mmHg)	120.160 ± 4.42	117.62 ± 2.90	123.91 ± 3.52
DBP (mmHg)	76.944 ± 4.11	74.68 ± 2.84	80.29 ± 3.35

**Table 4:** Comparison of obesity-related parameters between non-MetS and MetS PCOS subjects (independent samples t-test) Data are expressed as mean ± SD. Welch's correction was applied to the variables due to unequal variance. A p-value < 0.05 was considered statistically significant. BMI: body mass index; WHR: waist-to-hip ratio; MetS: metabolic syndrome

Variables	Non-MetS (n=86)	MetS (n=58)	t-statistics	p-value
Age (years)	27.18 ± 0.37	27.87 ± 0.62	Welch's t=0.952	0.344
BMI	24.34 ± 0.16	29.39 ± 0.34	Welch's t=13.276	<0.001
Waist (cm)	89.94 ± 0.60	94.94 ± 0.31	Welch's t=10.286	<0.001
Hip (cm)	98.99 ± 0.29	100.82 ± 0.21	Welch's t=5.015	<0.001
WHR	0.90 ± 0.005	0.96 ± 0.003	Welch t=8.472	<0.001

**Table 5:** Comparison of adipokines and endocrine variables between the non-MetS and MetS PCOS subjects (independent samples t-test) Data are expressed as mean ± SD. Welch's correction was applied to the variables due to unequal variance. A p-value < 0.05 was considered statistically significant. MetS: metabolic syndrome

Endocrinological Analytes	Non-MetS (n=86)	MetS (n=58)	t-statistics	p-value
Adiponectin (µg/mL)	12.35 ± 0.22	7.19 ± 0.26	Student's t=14.64	<0.001
Resistin (ng/mL)	4.78 ± 0.08	6.33 ± 0.10	Student's t=11.64	<0.001
Leptin (ng/mL)	10.15 ± 0.22	12.11 ± 0.22	Student's t=6.00	<0.001
Insulin (µg/mL)	9.02 ± 0.23	10.50 ± 0.16	Welch's t=5.08	0.029
Testosterone (ng/mL)	0.65 ± 0.01	0.69 ± 0.01	Welch’s t=2.36	0.019

**Table 6:** Comparison of biochemical parameters between the non-MetS and MetS PCOS subjects (independent samples t-test) Data are expressed as mean ± SD. A p-value < 0.05 was considered statistically significant. FBS: fasting blood sugar; HDL-C: high-density lipoprotein cholesterol; MetS: metabolic syndrome

Biochemical Analytes	Non-MetS (n=86)	MetS (n=58)	t-statistics	p-value
FBS (mg/dL)	86.51 ± 8.08	95.67 ± 7.55	6.84	<0.001
Triglyceride (mg/dL)	131.46 ± 9.63	164.60 ± 10.90	19.20	<0.001
Cholesterol (mg/dL)	153.71 ± 11.91	174.44 ± 11.76	10.29	<0.001
HDL-C (mg/dL)	39.04 ± 2.37	37.67 ± 1.83	-3.72	<0.001

A multinomial logistic regression model was developed to assess the role of adipokines and other factors in the progressive development of MetS among PCOS patients. MetS was kept as the dependent variable.

Three reference categories were formed: Category 1 comprises the presence of any three criteria for the diagnosis of MetS, Category 2 comprises the presence of any four criteria for the diagnosis of MetS, and Category 3 comprises full-blown MetS, where all five criteria for diagnosis were observable. The reference point was changed depending on the number of criteria fulfilled for MetS. Three reference points were made out of the three categories, and the analysis was performed with Category 2 as the reference point to have a bidirectional perspective of the problem. During the development of MetS, progression from Category 1 to Category 2, we observed that adipokines, anthropometric parameters, HDL-C, and BP significantly predict the disease progression. In contrast, TG and FBS failed as significant predictors.

The interplay of these variables completely changes as the progression moves from Category 2 to full-blown (Category 3) MetS. FBS, TG levels, and WC remain the only significant predictors during the later stages of MetS progression (Table 7).

**Table 7:** Multinomial regression analysis for assessing the role of adipokines as predictors for the development of MetS Model fit measures: deviance = 18.8, AIC (Akaike Information Criterion) = 58.8, R^2^McF = 0.921. The overall model test values are χ² = 219, df = 18, and p < 0.001. FBS: fasting blood sugar; HDL-C: high-density lipoprotein; SBP: systolic blood pressure; DBP: diastolic blood pressure

MetS Category Model	Predictor	Estimate	SE	Z	p-value
1–2	Intercept	28.728	0.0774	371.069	<0.001
	Waist (cm)	-76.885	0.6303	-121.975	<0.001
	Adiponectin (µg/mL)	-39.944	2.3901	-16.713	<0.001
	Resistin (ng/mL)	-12.908	0.4051	-31.866	<0.001
	Leptin (ng/mL)	-8.133	0.9415	-8.639	<0.001
	FBS (mg/dL)	1.643	6.6254	0.248	0.804
	Triglyceride (mg/dL)	5.524	11.3721	0.486	0.627
	HDL-C (mg/dL)	-11.508	3.3314	-3.454	<0.001
	SBP (mmHg)	23.649	4.1975	5.634	<0.001
	DBP (mmHg)	44.319	3.1562	14.042	<0.001
3–2	Intercept	-204.366	0.0140	-14567.680	<0.001
	Waist (cm)	0.842	0.3015	2.792	0.005
	Adiponectin (µg/mL)	0.214	0.3620	0.592	0.554
	Resistin (ng/mL)	0.937	0.8544	1.097	0.273
	Leptin (ng/mL)	-0.795	0.6159	-1.291	0.197
	FBS (mg/dL)	0.354	0.1334	2.656	0.008
	Triglyceride (mg/dL)	0.461	0.1274	3.618	<0.001
	HDL-C (mg/dL)	0.477	0.3835	1.243	0.214
	SBP (mmHg)	0.183	0.2545	0.720	0.472
	DBP (mmHg)	-0.209	0.3601	-0.580	0.562

## Discussion

The present study observed that 40.27% (n=58) of PCOS subjects had MetS out of the total study population. Ehrmann et al. (2006) estimated an overall prevalence of 33.4% of MetS in PCOS. They also observed the prevalence among different ethnic groups and found 26% in African Americans, 50% in Asians, 34% in Caucasians, 31% in Hispanics, and 43% in women with mixed ancestral origin [27]. Women with PCOS have high rates of T2DM and show the presence of risk factors for CVD. It has also been demonstrated that 71% of all PCOS have at least one component of MetS [28].

On phenotypic assessment, we observed that the non-PCO (O+HA) phenotype has a maximum number of patients with MetS (53, 91.38%), followed by complete PCOS (P+O+HA) (five, 10.87%). In the non-HA (P+O) and ovulatory phenotypes (P+HA), MetS was not observed. The non-PCO phenotype (O+HA) has the highest BMI and WC, which also indicates the role of abdominal obesity in the development of MetS. HA increases gene expression involved in lipogenesis, leading to fat accumulation, particularly in the abdominal cavity [29]. Additionally, insulin resistance, which is linked to compensatory hyperinsulinemia, stimulates the ovaries, resulting in increased androgen secretion [30]. Increased androgen increases fat accumulation, linking raised WC and BMI and the presence of MetS with the hyperandrogenic phenotype [31].

Increased WC values were found to be a constant feature of PCOS subjects with MetS. WC was shown to have a sensitivity of 97% when used independently for screening of MetS [15]. The mean BMI of our study population was 26.37, the mean BMI of the MetS group was 29.39, and that of the non-MetS group was 24.34. Cross-sectional studies examining the common characteristics of MetS and PCOS have demonstrated that the prevalence of MetS is higher in overweight or obese individuals with PCOS, with a subsequently significant correlation [32]. Dyslipidemia, along with hyperinsulinemia and hypertension, is related to obesity in PCOS rather than ovulatory function [33]. Our observations of differences in HDL and TG values between MetS and non-MetS also showed a strong correlation with BMI and WC.

Lower adiponectin concentration is associated with T2DM, obesity, dyslipidemia, and CVD. We observed that the mean concentration of adiponectin in the MetS group was significantly lower than that in the non-MetS group. Decreased adiponectin levels have been reported in PCOS, which may be the result of altered adipose tissue function and a difference in fat distribution in PCOS women, who exhibit more visceral fat [34].

Independent association of serum adiponectin with MetS in Chinese women with PCOS has been reported, and our study shows similar characteristics [35]. In an age- and gender-adjusted study, it was observed that the relationship between measures of obesity and adiponectin are negatively correlated [36]. PCOS generally coexists with obesity, but irrespective of BMI and the absence of additional weight, women with PCOS have increased WC and insulin resistance along with leptin resistance [37]. A study reported that where age and weight were matched between PCOS subjects and controls, there was a higher level of circulating leptin in the PCOS group. Significant elevation of leptin levels was seen in hyperinsulinemic, hyperandrogenemic, and obese subgroups [38]. We also found a difference in leptin levels between the MetS and non-MetS groups, and similar to other studies, our MetS group had higher fasting insulin and HA with elevated BMI.

Studies have indicated that obesity is the primary determinant for increased expression of resistin in PCOS, and it is also considered a marker of promoting angiogenesis [39]. Resistin levels were also found to be significantly correlated with components of MetS when compared to controls. It is also observed that this adipokine is linked with the severity of the syndrome [40]. In our study, we have found that circulating resistin was significantly high among PCOS patients with MetS. Resistin also correlates with plasma inflammatory chemokine concentration along with components of MetS in non-diabetic patients, indicating its role and involvement in the pathophysiology of MetS [25]. Resistin expression is predominantly observed in human omental and abdominal subcutaneous white adipocytes [41]. Central obesity is a key component of MetS. In our study, we have found a marked difference in WC between the MetS and non-MetS groups.

Some research also indicates that adipokine gene polymorphism can be one of the factors for altered circulating adipokine concentration. Boumaiza et al. (2012) have reported an association of resistin gene polymorphism with MetS parameters [42]. Li et al. (2015) have shown that adiponectin gene polymorphism is associated with its lower circulating concentration in the presence of MetS [43].

**Figure 3:**
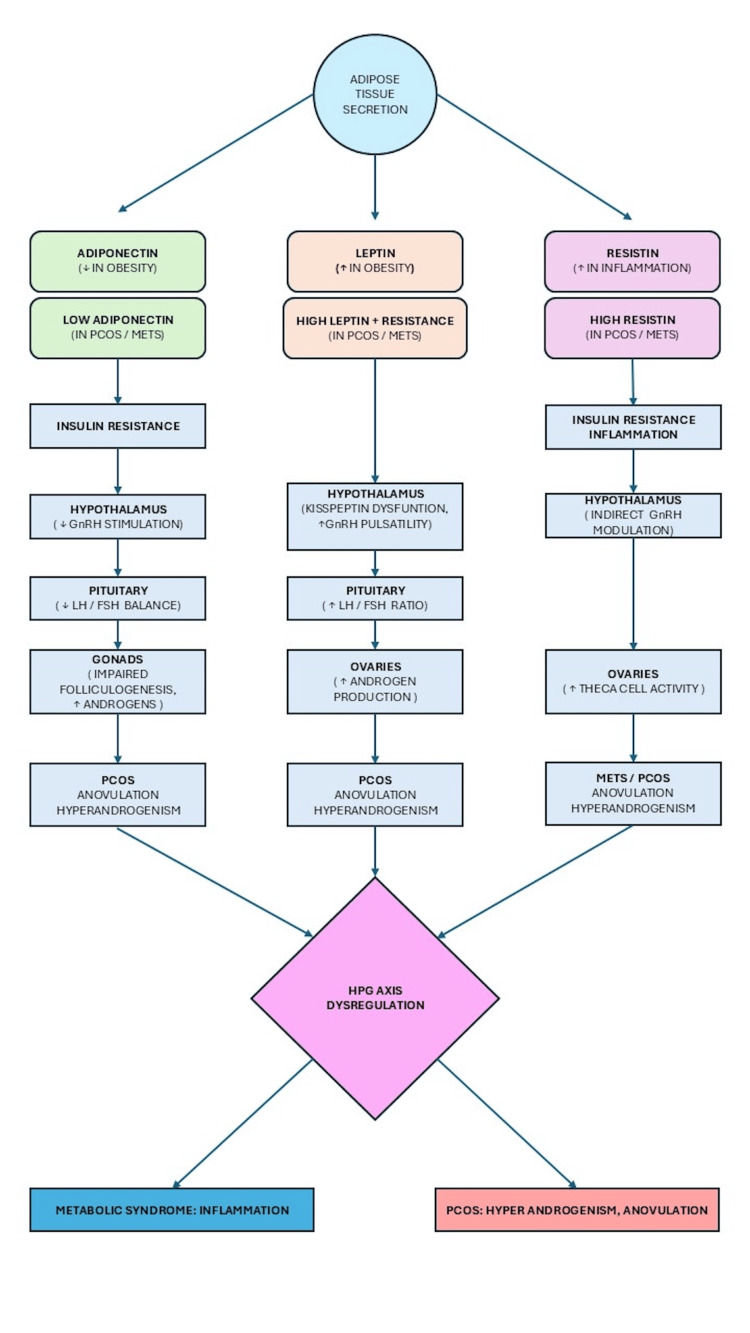
Effect of adipokines on the hypothalamus-pituitary-gonadal (HPG) axis

Leptin gene polymorphism is also associated with MetS and obesity [44]. The hypothalamus-pituitary-gonad (HPG) axis is key to linking the relationship between metabolic health and reproductive function and is closely regulated by the interactions of adipokines such as adiponectin, resistin, and leptin. Adiponectin receptors (AdipoR1 and AdipoR2) are present in hypothalamic GnRH hormone-secreting neurons and pituitary cells [45]. Obesity-induced hypoadiponectinemia leads to oversecretion of luteinizing hormone (LH), thus disturbing the LH/FSH (follicle-stimulating hormone) balance, which is a hallmark of the condition [46]. Although it is unknown how resistin directly affects the HPG axis, elevated resistin levels in PCOS potentially exacerbate insulin resistance, amplifying metabolic stress and androgen excess rather than directly altering gonadotropin dynamics [47]. Within the physiological limits, leptin stimulates GnRH release; however, excessive leptin disrupts the HPG axis, leading to disrupted gonadotropin pulsatility, which leads to androgen excess and anovulation (Figure 3) [48,49].

We divided the MetS group into three categories. Category 1 represented those who fulfilled three of the five criteria, Category 2 represented patients fulfilling four out of five criteria, and Category 3 comprised those with full-blown MetS, fulfilling all five criteria. Multiple logistic regressions were performed where the MetS category was chosen as the dependent variable and the indicators of MetS and adipokines as covariates. We kept Category 2 as the reference level to have a bidirectional view of MetS progression with higher numbers of altered parameters. We observed that in the progression from Category 1 to Category 2, adiponectin (β = -39.94, p < 0.001), resistin (β = -12.90, p < 0.001), and leptin (β = -8.133, p < 0.001) predicted the association significantly along with other indicators. Associations with FBS (β = 1.643, p = 0.804) and TG (β = 5.524, p = 0.627) were non-significant (Table 7).

Early stages of MetS development can be significantly associated with these altered adipokines without the presence of hyperglycemia and dyslipidemia. Various studies [50] have emphasized the usefulness of measuring adiponectin for the management of MetS. Studies also show the association of MetS risk with a decrease in adiponectin. As the individual component of MetS increases, there is a stepwise decrease in adiponectin level [51]. Alteration of adiponectin before the onset of hyperglycemia and dyslipidemia may be explained by the fact that, in spite of elevated expression of mRNA for adiponectin, it has been observed that there is a decrease in receptor expression and also deactivation of the PPAR-α/AMPK (AMP-activated protein kinase and peroxisome-activated receptor-α) pathway. This results in the downregulation of several genes involved in fatty acid oxidation and glucose metabolism [52].

Between Categories 2 and 3, it was observed that WC (β = 0.842, p < 0.01), FBS (β = 0.354, p < 0.01), and TG (β = 0.461, p < 0.001) significantly predicted the association. Between Categories 2 and 3, TG had the most significant association (Table 7). A meta-analysis by Lim et al. (2019) revealed an independent and strong association of insulin resistance and obesity with MetS in PCOS. It has been reported that proinflammatory adipokines (resistin) promote insulin resistance and influence liver function to increase hepatic glucose and TG production. The decrease in adiponectin further contributes to insulin sensitivity. There was a moderate correlation between HA and insulin resistance, but androgen did not correlate with any other metabolic components [5]. The study by Ehrmann et al. (2006) reported that elevated TG levels have a high predictive value for MetS. It was reported that 83% of women with a TG value of more than 150 mg/dL have the presence of at least two other components of MetS. They also observed that WC has a negative predictive value for MetS, and 96% of women with a WC of less than 88 cm did not have MetS [28]. The study of Arthur et al. (2012) examined how indicators of obesity and its related determinants predict MetS and influence its progression [53]. WHR significantly predicts metabolic risk followed by dyslipidemia in both pre- and post-menopausal women [54].

Limitations

The study employed a cross-sectional design and included participants from a single center who had symptoms of MetS. A longitudinal cohort may have been established to enhance understanding of the condition's progression. The generalizability of the result may be a concern from a single data source and can be addressed by a similar multicentric study. The study would become more empirical in character and provide a more comprehensive picture if genetic predisposition and polymorphism were evaluated. Future research could provide a more comprehensive understanding of how PCOS contributes to MetS and improve early diagnosis and management strategies for affected women.

## Conclusions

The observations of the present study suggest that the studied adipokines can significantly predict the progression of MetS from Category 1 to Category 2. In contrast, these progressions from Category 2 to Category 3 were better predicted by FBS and TG, along with WC, which emerged as significant predictors. The predictive significance of WC decreased moderately along with adipokines. Thus, the progression of MetS development can be significantly associated with these altered adipokines and may be used as an early diagnostic tool for the management of MetS in PCOS.
